# Convergent patterns of tissue-level distribution of elements in different tropical woody nickel hyperaccumulator species from Borneo Island

**DOI:** 10.1093/aobpla/plaa058

**Published:** 2020-11-13

**Authors:** Farida Abubakari, Jolanta Mesjasz-Przybyłowicz, Wojciech J Przybyłowicz, Antony van der Ent

**Affiliations:** 1 Centre for Mined Land Rehabilitation, Sustainable Minerals Institute, The University of Queensland, Sir James Foots Building, Brisbane, QLD, Australia; 2 Department of Botany and Zoology, Stellenbosch University, Private Bag, Matieland, South Africa; 3 AGH University of Science and Technology, Faculty of Physics & Applied Computer Science, al. Mickiewicza, Kraków, Poland; 4 Universite de Lorraine – INRA, Laboratoire Sols et Environnement, UMR, Vandoeuvre-les-Nancy, France

**Keywords:** Elemental distribution, elemental maps, hyperaccumulator, micro-PIXE, nuclear microprobe, X-ray microanalysis

## Abstract

The Malaysian state of Sabah on the Island of Borneo has recently emerged as a global hotspot of nickel hyperaccumulator plants. This study focuses on the tissue-level distribution of nickel and other physiologically relevant elements in hyperaccumulator plants with distinct phylogenetical affinities. The roots, old stems, young stems and leaves of *Flacourtia kinabaluensis* (Salicaceae), *Actephila alanbakeri* (Phyllanthaceae), *Psychotria sarmentosa* (Rubiaceae) and young stems and leaves of *Glochidion brunneum* (Phyllanthaceae) were studied using nuclear microprobe (micro-PIXE and micro-BS) analysis. The tissue-level distribution of nickel found in these species has the same overall pattern as in most other hyperaccumulator plants studied previously, with substantial enrichment in the epidermal cells and in the phloem. This study also revealed enrichment of potassium in the spongy and palisade mesophyll of the studied species. Calcium, chlorine, manganese and cobalt were found to be enriched in the phloem and also concentrated in the epidermis and cortex of the studied species. Although hyperaccumulation ostensibly evolved numerous times independently, the basic mechanisms inferred from tissue elemental localization are convergent in these tropical woody species from Borneo Island.

## Teaser

Hyperaccumulators are rare plants that accumulate trace elements to extreme concentrations in their living shoots. This study focuses on the tissue-level distribution of nickel and other physiologically relevant elements in four different nickel hyperaccumulator plants from the Malaysian state of Sabah on the Island of Borneo. Even though nickel hyperaccumulation has ostensibly evolved numerous times independently in distant phylogenetic lineages in different areas around the world, the physiological mechanisms, as inferred from elemental localization, are convergent in the tropical woody species that were studied from Borneo Island.

## Introduction

Plants require some trace elements in minor quantities (e.g. Mn, Fe, Ni, Zn) for healthy growth, whereas excess of these can lead to toxicity symptoms (DalCorso *et al.* 2014). Other elements, such as Na, Al, Si and Co, although not essential, are known to be beneficial to some plant species (Pilon-Smits *et al.* 2009). Macronutrients (Mg, P, S, K and Ca) are needed for basic plant metabolism and to protect plants from various abiotic and biotic stresses (Shanker and Venkateswarlu 2011; Rowley *et al.* 2012; Morgan and Connolly 2013). Hyperaccumulators are plants that accumulate trace elements to extreme concentrations (e.g. Ni > 1000 μg g^−1^) in their living shoots (Reeves 2003; van der Ent *et al.* 2013a). There are currently >500 nickel hyperaccumulator plant species known globally, with the greatest number of species recorded in Cuba, New Caledonia and the Mediterranean Region (Baker and Brooks 1988; Reeves 2003; Reeves *et al.* 2017). At a global scale, the most common families of Ni hyperaccumulators in tropical regions are the Phyllanthaceae, Rubiaceae and Salicaceae (Reeves 2006). Nickel hyperaccumulator plants have the potential to be used in phytomining, an environmentally sustainable ‘green’ technology to produce Ni (Chaney 1983; Chaney *et al.* 1998, 2007; van der Ent *et al.* 2013b). In a phytomining operation, hyperaccumulator plants are grown on ultramafic soils, followed by harvesting, drying and incineration of the above-ground biomass to generate a commercial high-grade Ni bio-ore (Brooks and Robinson 1998; Chaney *et al.* 2007, 2018; van der Ent *et al.* 2013b, 2017c; Bani *et al.* 2015).

The ultramafic soils of the Malaysian state of Sabah on the Island of Borneo are renowned for high species richness (van der Ent *et al.* 2014), with over 5000 plant species known from the <1200 km^2^ Kinabalu Park area (Beaman 2005) and 2854 plant species in 742 genera and 188 families recorded from the ultramafic soils in Kinabalu Park (van der Ent *et al.* 2014, 2016). In Sabah, 28 Ni hyperaccumulator species in 10 families and 17 genera are now known (van der Ent *et al.* 2019b), and most Ni hyperaccumulator species are from the order Malpighiales, predominantly in the families Phyllanthaceae, Salicaceae and Violaceae (van der Ent *et al.* 2019a, b).

**Figure 1.**
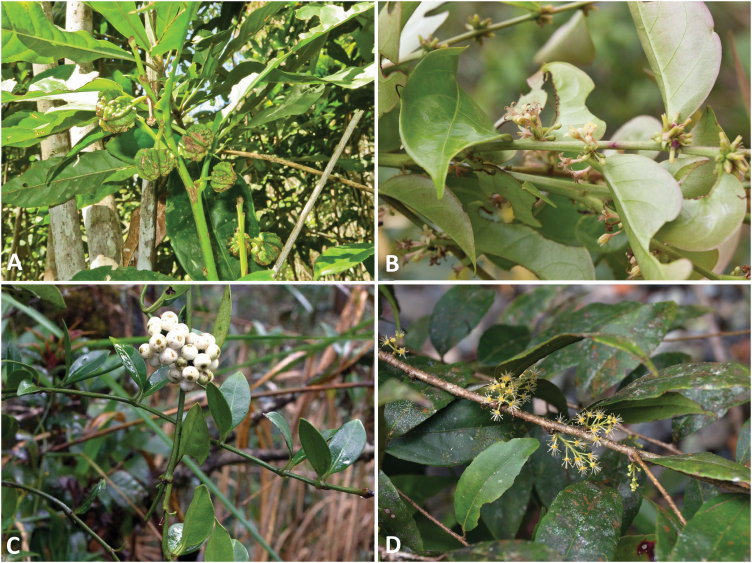
Foliage of the Ni hyperaccumulator plants species studied. (A) *Actephila alanbakeri*, (B) *Glochidion brunneum*, (C) *Psychotria sarmentosa* and (D) *Flacourtia kinabaluensis*.

*Actephila alanbakeri* and *Glochidion brunneum* (Fig. 1) are both members of the Phyllanthaceae family, which globally has the greatest numbers of Ni hyperaccumulating taxa (Reeves 2003) together with the closely related families Buxaceae (genus: *Buxus*) and Euphorbiaceae (genus: *Leucocroton*). *Glochidion brunneum* is widespread in Indonesia, Malaysia and the Philippines. In contrast, *A. alanbakeri* is a local endemic known from just two populations in Sabah near Kinabalu Park and Malawali Island. *Glochidion brunneum* is a medium-sized (up to 10 m tall) understorey tree of lowland rainforest. It can accumulate up to 6200 µg g^−1^ foliar Ni (van der Ent *et al.* 2015a). *Actephila alanbakeri* is a small (up to 3 m tall) woody shrub of disturbed habitats on eroded soils (Hypermagnesian Cambisols). This species may accumulate up to 14 700 µg g^−1^ foliar Ni (van der Ent *et al.* 2015b). *Psychotria sarmentosa* is a member of Rubiaceae family (Fig. 1) and is a climber that occurs in lowland forest, mainly in disturbed areas. The species is widespread in Indonesia, Malaysia and the Philippines. It is a strong Ni hyperaccumulator which can attain up to 24 200 µg g^−1^ foliar Ni (van der Ent *et al.* 2015a) (Fig. 1). *Flacourtia kinabaluensis* is a member of the Salicaceae family. It is a small tree (up to 8–12 m tall) that primarily occurs in riparian habitats. This species is a local endemic of the Kinabalu Park region of Sabah, Malaysia. It accumulates up to 7300 foliar µg g^−1^ Ni (van der Ent *et al.* 2015a) (Fig. 1).

Previous studies regarding the distribution and chemical speciation of Ni in hyperaccumulators from Borneo (Sabah) have focussed on *Rinorea* cf. *bengalensis*, *R.* cf. *javanica* (Violaceae), *Phyllanthus balgooyi*, *P. rufuschaneyi* (previously designated as *P.* cf. *securinegoides*) and *Glochidion* cf*. sericeum* (Phyllanthaceae) using nuclear microprobe (micro-proton-induced X-ray emission (micro-PIXE)) analysis with backscattering spectrometry (BS). Additionally, Ni distribution in these species has been studied with the use of synchrotron X-ray Fluorescence Microscopy (XFM) and X-ray Absorption Spectroscopy (XAS) techniques (Mesjasz-Przybyłowicz *et al.* 2016a; van der Ent *et al.* 2017a, 2018, 2020). The results showed that Ni was primarily concentrated in the epidermal areas of the leaves, and Ni in roots and stems of all three species was exceptionally enriched in the phloem. Nickel distribution in leaves, however, varies by species. In *P. balgooyi* the highest foliar Ni concentration was in the phloem, but in *P. rufuschaneyi* and *R. bengalensis* the highest foliar Ni concentration was in the epidermis and spongy mesophyll (*R.* cf. *bengalensis*). *Phyllanthus balgooyi* was unusual with extreme accumulation of Ni in the phloem with up to 169 g kg^−1^ Ni in the phloem sap (van der Ent and Mulligan 2015). This phloem sap concentration is second only to the New Caledonian tree *Pycnandra acuminata*, which may contain up to 257 g kg^−1^ Ni in the latex (Jaffré *et al.* 1976). The chemical form of Ni was consistently associated with citrate and did not differ between the species in all of the tissues (roots, phloem and leaves) nor in the transport liquids (xylem and phloem) (van der Ent *et al.* 2017a). In *Phyllanthus serpentinus* and *Psychotria gabriellae* from New Caledonia, Ni-malate was reported as the dominant chemical form of Ni within the plant cells (Kersten *et al.* 1980), whereas citrate was found as the major ligand in several other hyperaccumulator plant species, e.g. *P. acuminata*, *Hybanthus caledonicus* (Lee *et al.* 1977, 1978; Kersten *et al.* 1980).

The current research aims to expand the knowledge base on tropical Ni hyperaccumulator plant species by investigating a number of species originating from different families: Rubiaceae (*P. sarmentosa*), Salicaceae (*F*. *kinabaluensis*) and from different genera from the Phyllanthaceae family (*G. brunneum*, *A. alanbakeri*) using micro-PIXE analysis. Specifically, the tissue-level distribution of Ni and other physiologically relevant elements in these species will be compared with information available for the *Rinorea* spp. and *Phyllanthus* spp. studied previously. Through this analysis we aimed to establish whether patterns of tissue-level elemental distribution are different in phylogenetically distant hyperaccumulator species, and hence whether basic underlying mechanisms of Ni hyperaccumulation may be distinct or similar.

## Materials and Methods

### Collection and bulk analysis of plant tissue samples

Young plants of *F. kinabaluensis*, *A. alanbakeri*, *G. brunneum* and *P. sarmentosa* were collected in their natural habitats in and near Kinabalu Park in Sabah (Malaysia) on the island of Borneo. These wild-collected plant specimens were subsequently potted in the nursery of the ‘Hyperaccumulator Botanical Garden’ at Monggis substation of Kinabalu Park and cultivated there for ~1 year. Individuals of *G. brunneum* were growing naturally near the nursery.

Tissue samples including roots, old stems, young stems and leaves of *F. kinabaluensis*, *A. alanbakeri*, *P. sarmentosa* and young stems and leaves of *G. brunneum* grown in cultivation at the Hyperaccumulator Botanical Garden were harvested. Branches, fruits and berries of *P. sarmentosa* and phloem of *A. alanbakeri* were also harvested. Root tissues were thoroughly washed with water to remove potentially particulate (soil) contamination. The plant tissue samples were cut out with a surgical stainless-steel knife directly from the living plants. The samples collected for micro-PIXE analysis were immediately flash-frozen in the field using a cold mirror technique in which the samples were pressed between a large block of copper metal cooled by liquid nitrogen (−196 °C) and a second block of copper attached to a Teflon holder. This ensured extremely fast freezing of the plant tissue samples to prevent cellular damage by ice crystal formation. The samples were then wrapped in aluminium foil and transported in a cryogenic container directly to iThemba LABS in South Africa for analysis. Phloem samples were collected by stripping sections of this tissue from beneath the bark.

Plant tissue subsamples were dried at 70 °C for 5 days in a dehydrating oven for bulk elemental analysis. The dried plant tissue samples were subsequently ground, and a 300-mg fraction was digested using 5 mL concentrated nitric acid (70 %) in a digestion microwave oven (Milestone Start D) for a 45-min programme, and after cooling diluted to 30 mL with ultrapure water. The samples were then analysed by ICP-AES (Varian Vista Pro II) for Na, Mg, Al, P, S, K, Ca, Cr, Mn, Fe, Co, Ni, Cu, and Zn.

### Nuclear microprobe elemental analysis of plant tissues

Specimens were removed from the LN_2_ storage container and freeze-dried in a Leica EM CFD Cryosorption Freeze Dryer (Leica Microsystems AG, Austria). The freeze-drying process followed a long, 208-h programmed cycle to prevent shrinkage of the tissues. Freeze-dried plant tissues were then hand-cut with a steel razor blade and mounted on specimen holders covered with 0.5 % Formvar film and lightly coated with carbon to prevent charging. Elemental microanalyses were performed using the nuclear microprobe at the Materials Research Department, iThemba LABS, South Africa. The facility and methodology of measurements of biological materials have been reported elsewhere in detail (Prozesky *et al.* 1995; Przybyłowicz *et al.* 1999, 2005).

Nuclear microprobe elemental analysis uses a proton beam of 3 MeV energy, provided by a 6-MV single-ended Van de Graaff accelerator. The proton beam was focussed to a 3 × 3 μm^2^ spot and raster-scanned over the areas of interest, using square or rectangular scan patterns with a variable number of pixels (up to 128 × 128). Proton current was restricted to 100–150 pA to minimize specimen beam damage. Proton-induced X-ray emission and proton BS were used simultaneously. Proton-induced X-ray emission spectra were registered with a Si(Li) detector manufactured by PGT (30 mm^2^ active area and 8.5 µm Be window) with an additional 125 µm Be layer as an external absorber. The effective energy resolution of the PIXE system (for the Mn Kα line) was 160 eV, measured for individual spectra. The detector was positioned at a take-off angle of 135° and a working distance of 24 mm. The X-ray energy range was set between 1 and 40 keV. Backscattering spectrometry spectra were recorded with an annular Si surface barrier detector (100 μm thick) positioned at an average angle of 176°. Data were acquired in the event-by-event mode. The normalization of results was performed using the integrated beam charge, collected simultaneously from a Faraday cup located behind the specimen and from the insulated specimen holder. The total accumulated charge per scan varied from 0.51 to 3.82 µC.

The concentration and distribution of Si, P, S, Cl, K, Ca, Mn, Fe, Co, Ni, Cu, Zn, Br, Rb and Sr were quantified in the freeze-dried plant tissues of *F. kinabaluensis*, *A. alanbakeri, G. brunneum* and *P. sarmentosa*. These quantitative results were obtained by a standardless method using GeoPIXE II software package (Ryan *et al.* 1990a, b; Ryan 2000). The error estimates are extracted from the error matrix generated in the fit, and the minimum detection limits are calculated using the Currie equation (Currie 1968). The detailed calibration of detector efficiency, the thicknesses of selectable X-ray-attenuating filters and studies on the accuracy and precision have been reported elsewhere (van Achterbergh *et al.* 1995). The procedure reported there was used for the PGT Si(Li) detector used in the present study. The calibration of the analytical system was tested by measurements of standards (pure elements and synthetic glasses with known quantities of selected minor elements), the X-ray peaks of which cover practically the whole measurable energy range. Quantitative elemental mapping was performed using Dynamic Analysis method (Ryan and Jamieson 1993; Ryan *et al.* 1995; Ryan 2000). This method generates elemental images, which are (i) overlap-resolved, (ii) with subtracted background and (iii) quantitative, i.e. accumulated in μg g^−1^ dry weight units. Maps were complemented by data extracted from arbitrarily selected micro-areas within scanned plant tissue. Particle-induced X-ray emission and BS spectra were employed to obtain average concentrations from these micro-areas using a full non-linear deconvolution procedure to fit PIXE spectra (Ryan *et al.* 1990a, b), with matrix corrections based on thickness and matrix composition obtained from the corresponding BS spectra, fitted with a RUMP simulation package (Doolittle 1986) with non-Rutherford cross-sections for C, O, N.

### Electron microscopy of freeze-dried plant tissues

Freeze-dried leaf samples (24 h at −80 °C) were sputter-coated with carbon (25 nm) and mounted on stubs. The samples were then imaged with scanning electron microscopy (SEM) on a JEOL JSM-6610.

## Results

### Bulk chemistry of the studied hyperaccumulator plants

The results of the ICP-AES in plant tissues of *A. alanbakeri*, *G. brunneum*, *P. sarmentosa* and *F. kinabaluensis* are shown in Tables 1 and 2. The mean foliar Ni concentration in *A. alanbakeri* was 4500 µg g^−1^ (range from 280 to 14 700 µg g^−1^), 20 500 µg g^−1^ in *P. sarmentosa* (range from 9500 to 29 600 µg g^−1^), 770 µg g^−1^ in *F. kinabaluensis* and 3900 µg g^−1^ in *G. brunneum* (range from 2180 to 5540 µg g^−1^) (Table 1). In the roots Ni concentration was 970 µg g^−1^ in *A. alanbakeri* and 1090 µg g^−1^ in *P. sarmentosa*. In the old stems its concentration was between 3100 and 8430 µg g^−1^ in *P. sarmentosa* (mean value 6050 µg g^−1^), while in the young stems the ranges of concentrations were from 580 to 1700 µg g^−1^ in *A. alanbakeri* (mean value 1030 µg g^−1^) and from 6200 to 8800 µg g^−1^ in *P. sarmentosa* (mean value 7400 µg g^−1^). The mean foliar concentration of K was 17 100 µg g^−1^ in *A. alanbakeri* (range from 8800 to 22 400 µg g^−1^), 2880 µg g^−1^ in *P. sarmentosa* (range from 1260 to 4620 µg g^−1^), 14 000 µg g^−1^ in *F. kinabaluensis* and 5060 µg g^−1^ in *G. brunneum* (range from 4130 to 6280 µg g^−1^) (Table 2). *Actephila alanbakeri*, *P. sarmentosa* and *F. kinabaluensis* had foliar Ca concentrations ranging between 1520 and 11 300 µg g^−1^ (mean value 5720 µg g^−1^), between 2600 and 13 500 µg g^−1^ (mean value 6900 µg g^−1^) and 10 500 µg g^−1^, respectively, and between 4170 and 5300 µg g^−1^ in *G. brunneum* (mean value 4720 µg g^−1^). Foliar Mn concentration in *A. alanbakeri* ranged between 50 and 530 (mean value 180 µg g^−1^), whereas in *P. sarmentosa* it was between 680 and 1550 µg g^−1^ (mean value 1100 µg g^−1^), between 80 and 140 µg g^−1^ in *G. brunneum* (mean value 110 µg g^−1^) and 90 µg g^−1^ in *F. kinabaluensis*. Values for Cr, Fe, Co, Cu and Zn were low in the leaves of all of the studied species (Table 1).

**Table 1.** Concentrations of beneficial and trace elements in plant tissues in *Actephila alanbakeri*, *Glochidion brunneum*, *Psychotria sarmentosa* and *Flacourtia kinabaluensis* (values as ranges and [means] in µg g^−1^ dry weight) analysed by ICP-AES.

Species	*n*	Na	Al	Cr	Mn	Fe	Co	Ni	Cu	Zn
**Leaves**										
*Actephila alanbakeri*	6	30–910 [305]	15–60 [45]	3.0–30 [20]	50–530 [180]	40–100 [80]	10–55 [30]	280–14 700 [4500]	3.0–25 [15]	20–100 [50]
*Psychotria sarmentosa*	5	90–2400 [1480]	165–3770 [1200]	30–140 [95]	680–1550 [1100]	30–125 [60]	4.0–15 [10]	9500–29 600 [20 500]	4.0–15 [5]	50–100 [75]
*Flacourtia kinabaluensis*	1	220	50	60	90	70	85	770	25	50
*Glochidion brunneum*	3	180–570 [395]	30–60 [40]	5–10 [8]	80–140 [110]	10–65 [30]	20–40 [30]	2180–5540 [3900]	2 [2]	10–30 [20]
**Branches**										
*Psychotria sarmentosa*	1	1050	65	15	40	10	10	4820	5	30
**Fruit**										
*Psychotria sarmentosa*	1	850	430	10	290	25	10	3920	10	20
**Berries**										
*Psychotria sarmentosa*	1	95	70	10	50	10	10	6820	5	40
**Wood**										
*Actephila alanbakeri*	1	200	5	5	40	15	10	270	2	10
**Young stems**										
*Actephila alanbakeri*	3	240–390 [300]	5–110 [10]	1.0–2.0 [2.0]	70–290 [210]	10–30 [20]	5.0–10 [10]	580–1700 [1030]	2.0–3.0 [2.5]	20–70 [40]
*Psychotria sarmentosa*	3	1060–3150 [1960]	110–190 [140]	30–50 [40]	100–270 [190]	15–30 [20]	6.0–9.0 [7.5]	6200–8800 [7400]	3.0–6.00 [5.00]	20–90 [50]
**Old stems**										
*Psychotria sarmentosa*	3	220–1510 [1050]	30–320 [135]	20–170 [80]	30–220 [150]	15–20 [20]	10–15 [10]	3100–8430 [6050]	3.0–10 [4.50]	20–30 [20]
**Phloem**										
*Actephila alanbakeri*	1	250	30	2	350	50	15	2500	4	120
**Roots**										
*Actephila alanbakeri*	1	40	65	15	30	300	8	970	2	40
*Psychotria sarmentosa*	1	1500	405	20	70	270	8	1090	5	15

**Table 2.** Concentrations of macroelements in plant tissues in *Actephila alanbakeri*, *Glochidion brunneum*, *Psychotria sarmentosa* and *Flacourtia kinabaluensis* (values as ranges and [means] in µg g^−1^ dry weight) analysed by ICP-AES.

Species	*n*	Mg	P	S	K	Ca
**Leaves**						
*Actephila alanbakeri*	6	1280–6500 [4100]	300–1440 [852]	610–2770 [1770]	8800–22 400 [17 100]	1520–11 300 [5720]
*Psychotria sarmentosa*	5	4380–8100 [6000]	130–600 [330]	1360–1900 [1700]	1260–4620 [2880]	2600–13 500 [6900]
*Flacourtia kinabaluensis*	1	5400	810	2100	14 000	10 500
*Glochidion brunneum*	3	1320–2710 [2160]	160–210 [190]	530–700 [640]	4130–6280 [5060]	4170–5300 [4720]
**Branches**						
*Psychotria sarmentosa*	1	490	65	370	2430	850
**Fruit**						
*Psychotria sarmentosa*	1	3370	530	1040	5420	3960
**Berries**						
*Psychotria sarmentosa*	1	3720	325	515	7800	2660
**Wood**						
*Actephila alanbakeri*	1	520	270	290	1670	200
**Young stems**						
*Actephila alanbakeri*	3	1590–3460 [2620]	795–1210 [1060]	410–970 [680]	7230–10 600 [8880]	1280–6770 [3410]
*Psychotria sarmentosa*	3	1000–2090 [1500]	85–410 [210]	840–1260 [1010]	3710–6710 [5000]	1880–5560 [3590]
**Old stems**						
*Psychotria sarmentosa*	3	440–2440 [1150]	40–250 [140]	370–550 [460]	575–6530 [2730]	570–3540 [2000]
**Phloem**						
*Actephila alanbakeri*	1	4260	290	840	12 800	2090
**Roots**						
*Actephila alanbakeri*	1	1260	350	215	3900	310
*Psychotria sarmentosa*	1	650	110	380	910	1370

### Nuclear microprobe microanalyses of the studied hyperaccumulator plants

The results of the nuclear microprobe analysis in anatomical regions of the roots, old stems, young stems and leaves are shown in Tables 3–6, Figs 2–5 and **Supporting Information—Figs S1****–****S5**.

**Table 3.** Average elemental concentrations (micro-PIXE, µg g^−1^ dry weight) in the sections of the roots of *Actephila alanbakeri*, *Psychotria sarmentosa* and *Flacourtia kinabaluensis* (values as ranges and [mean values]).

*n*	Si	P	S	Cl	K	Ca	Cr	Mn	Fe	Co	Ni	Cu	Zn
*Actephila alanbakeri*													
3	4810–9830 [6060]	330–625 [515]	1890–3060 [2317]	3060–6300 [4863]	9460–14 800 [12 553]	345–890 [585]	30–40 [33]	30–55 [45]	770–1370 [1077]	6–20 [12]	100–370 [253]	3.5–4.5 [4]	40–70 [53]
***Psychotria sarmentosa***													
2	1710–9200 [5455]	200–220 [210]	2670–9800 [6235]	750–4070 [2410]	3620–4600 [4110]	900–2030 [1465]	1–40 [20]	10–30 [20]	120–1340 [730]	1–15 [7.5]	50–100 [75]	<2.1	6.5–20 [13.3]
***Flacourtia kinabaluensis***													
3	1640–9500 [4900]	160–250 [200]	480–11 700 [4300]	670–1780 [1120]	5600–9110 [7170]	740–1300 [1030]	7–85 [39]	6–65 [37]	250–1580 [820]	6–15 [9]	70–190 [120]	<3.5	10–30 [20]

**Table 4.** Average elemental concentrations (micro-PIXE, µg g^−1^ dry weight) in the sections of the old stems of *Actephila alanbakeri*, *Psychotria sarmentosa* and *Flacourtia kinabaluensis.* For single measurements errors of analysis (± SD) are shown in brackets. For *n* > 1 the results are shown as ranges and [mean values].

*n*	Si	P	S	Cl	K	Ca	Cr	Mn	Fe	Co	Ni	Cu	Zn
***Actephila alanbakeri***													
1	4070 (425)	380 (30)	620 (30)	1670 (20)	11 100 (50)	1800 (30)	15 (1.0)	60 (2)	500 (10)	3 (3)	700 (20)	3.5 (1.0)	90 (3)
*Psychotria sarmentosa*													
1	870 (250)	1050 (80)	1310 (54)	30 000 (270)	27 400 (170)	2440 (70)	30 (1)	20 (5)	80 (7)	<5	250 (20)	5 (2)	10 (1)
*Flacourtia kinabaluensis*													
2	5100–13 100 [9100]	310–417 [364]	440–1970 [1205]	520–540 [530]	7720–8230 [7975]	2340–2960 [2650]	5.5–40 [23]	15–100 [58]	410–1520 [965]	4–14 [9]	100–500 [300]	<3	20–35 [28]

**Table 5.** Average elemental concentrations (micro-PIXE, µg g^−1^ dry weight) in the sections of the young stems of *Actephila alanbakeri*, *Glochidion brunneum*, *Psychotria sarmentosa* and *Flacourtia kinabaluensis*. Errors of analysis (+ SD) are shown in brackets.

Si	P	S	Cl	K	Ca	Cr	Mn	Fe	Co	Ni	Cu	Zn
***Actephila alanbakeri***												
4420 (390)	1620 (130)	2000 (110)	1600 (40)	11 700 (80)	2950 (30)	14.5 (1)	155 (6)	770 (15)	3 (3)	1160 (30)	5 (1)	230 (6)
***Glochidion brunneum***												
140 (140)	350 (55)	1740 (40)	13 300 (80)	21 400 (90)	1540 (50)	<1.5	30 (4)	4 (3)	<7	175 (10)	4.5 (0.5)	9.0 (0.5)
***Psychotria sarmentosa***												
1100 (115)	510 (40)	1850 (25)	6900 (30)	13 500 (80)	1370 (30)	95 (3)	15 (5)	65 (5)	<3	190 (10)	2.0 (0.5)	10 (1)
***Flacourtia kinabaluensis***												
1010 (100)	640 (35)	2020 (80)	700 (40)	13 600 (80)	2570 (50)	<2.2	15 (1.0)	90 (5)	<5	100 (10)	<3.5	30 (2)

**Table 6.** Average elemental concentrations (micro-PIXE, µg g^−1^ dry weight) in the sections of the leaves of *Actephila alanbakeri*, *Glochidion brunneum*, *Psychotria sarmentosa* and *Flacourtia kinabaluensis*. For single measurements errors of analysis (+ SD) are shown in brackets. For *n* > 1 the results are shown as ranges and [mean values].

*n*	Si	P	S	Cl	K	Ca	Cr	Mn	Fe	Co	Ni	Cu	Zn
***Actephila alanbakeri***													
2	190–480 [2135]	220–1000 [610]	580–1090 [835]	4200–4620 [4410]	12 800–15 800 [14 300]	700–6800 [3750]	0.4–11 [5]	90–390 [240]	20–590 [305]	0.2–7 [4]	270–740 [505]	<5	15–61 [38]
***Glochidion brunneum***													
1	2420 (300)	<85	1170 (40)	17 300 (100)	38 200 (170)	3340 (100)	<3	110 (10)	25 (10)	<20	1060 (60)	<5	20 (2)
***Psychotria sarmentosa***													
3	740–1850 [1330]	300–670 [490]	1250–2500 [2070]	12 400–16 400 [14 030]	2300–7800 [4560]	690–1970 [1200]	1.1–40 [20]	25–120 [70]	80–200 [140]	<2	60–250 [170]	<3.5	5.5–45 [22]
***Flacourtia kinabaluensis***													
3	410–2780 [1720]	250–280 [263]	400–1250 [690]	1100–4300 [2300]	2650–10 300 [5330]	900–3470 [1770]	0.4–6 [2]	4–15 [10]	180–250 [210]	2–15 [7]	85–360 [195]	<5	8–45 [21]

**Figure 2.**
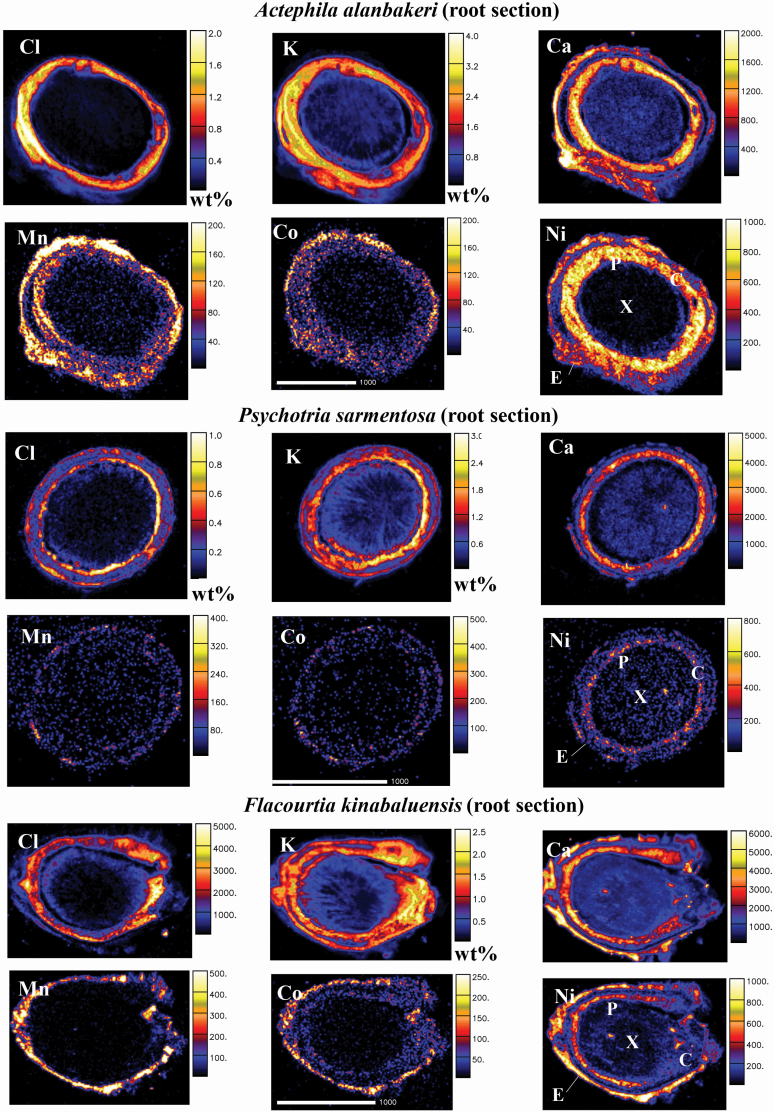
Micro-PIXE quantitative elemental maps of root cross-sections of *Actephila alanbakeri*, *Psychotria sarmentosa* and *Flacourtia kinabaluensis*. Concentration scale in wt% dry weight or in μg g^−1^ dry weight. Abbreviations of anatomical features: C, cortex; E, epidermis; P, phloem; and X, xylem.

**Figure 3.**
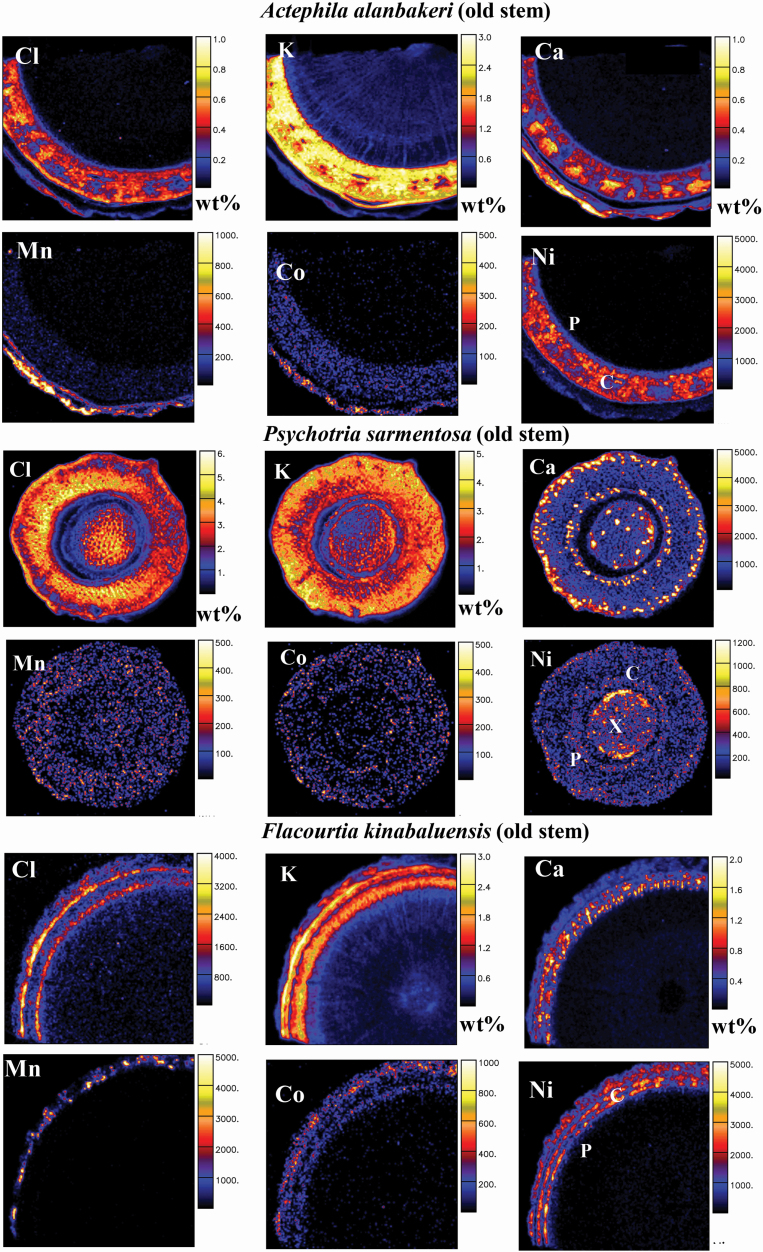
Micro-PIXE quantitative elemental maps of old stem cross-sections of *Actephila alanbakeri*, *Psychotria sarmentosa* and *Flacourtia kinabaluensis*. Concentration scale in wt% dry weight or in μg g^−1^ dry weight. Abbreviations of anatomical features: C, cortex; P, phloem; and X, xylem.

**Figure 4.**
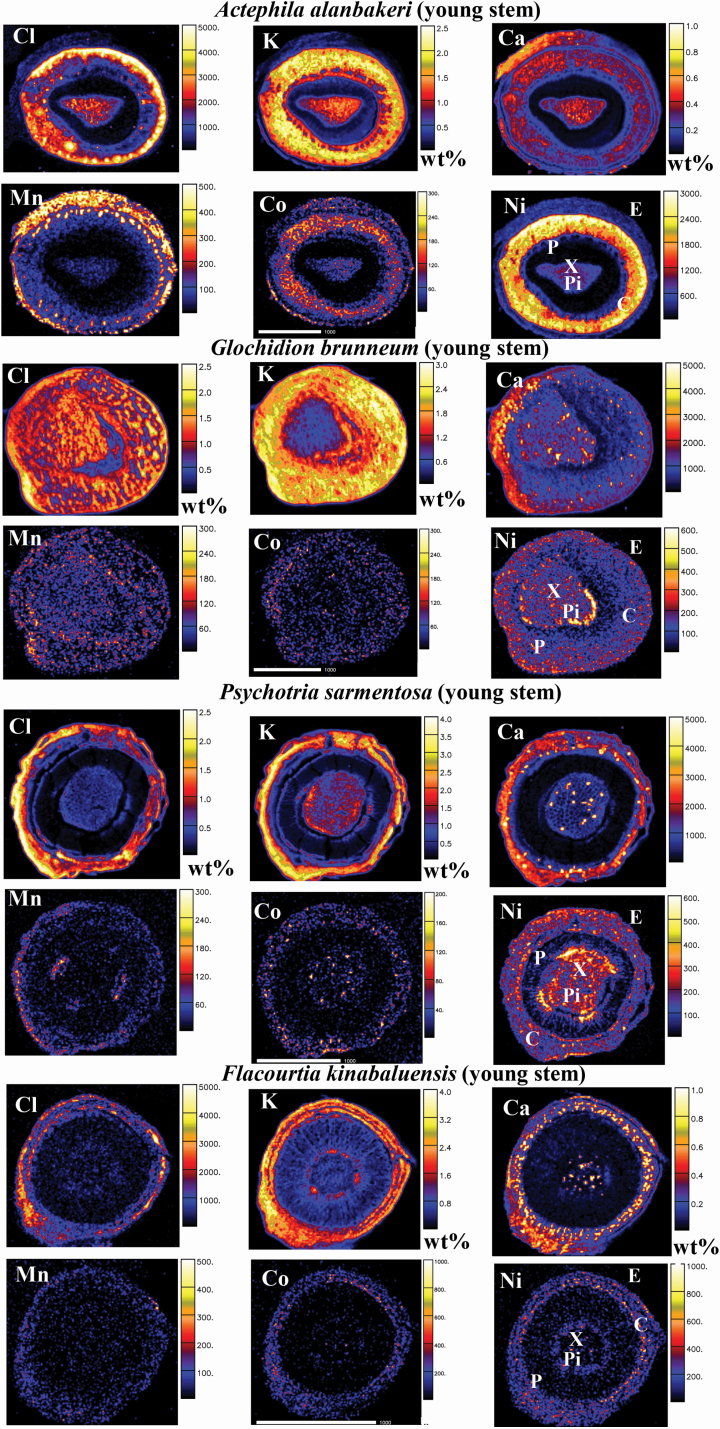
Micro-PIXE quantitative elemental maps of young stem cross-sections of *Actephila alanbakeri*, *Glochidion brunneum*, *Psychotria sarmentosa* and *Flacourtia kinabaluensis*. Concentration scale in wt% dry weight or in μg g^−1^ dry weight. Abbreviations of anatomical features: C, cortex; E, epidermis; Pi, pith; P, phloem; and X, xylem.

**Figure 5.**
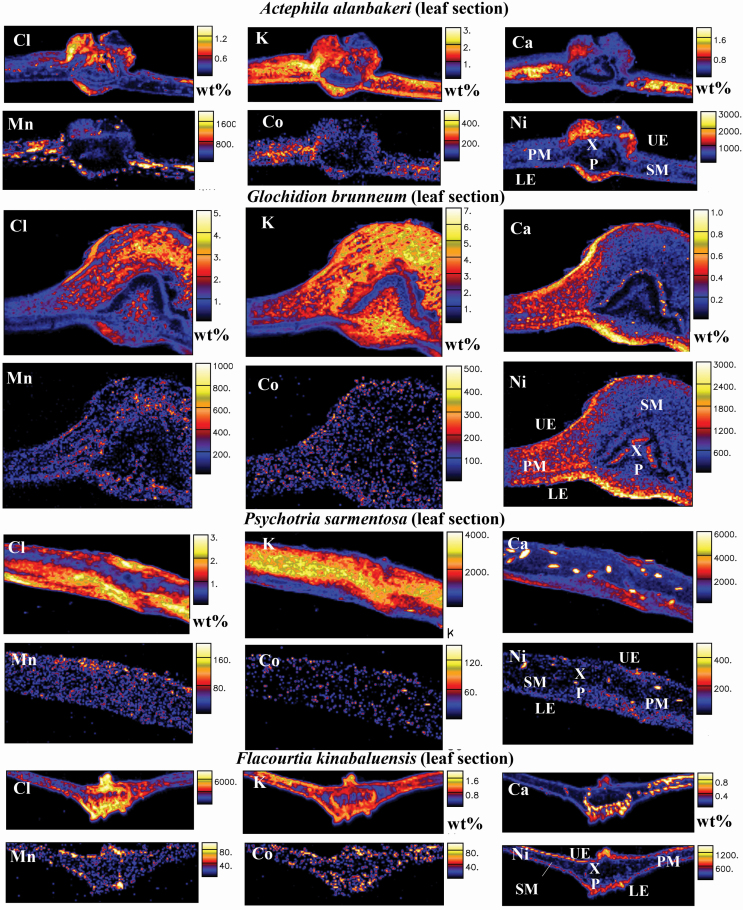
Micro-PIXE quantitative elemental maps of leaf cross-sections of *Actephila alanbakeri*, *Glochidion brunneum*, *Psychotria sarmentosa* and *Flacourtia kinabaluensis*. Concentration scale in wt% dry weight or μg g^−1^ dry weight. Abbreviations of anatomical features: UE, upper epidermis; LE, lower epidermis; PM, palisade mesophyll; SM, spongy mesophyll; P, phloem; and X, xylem.

#### Roots.

The concentrations of Ni in roots of *A. alanbakeri* ranged between 100 and 370 µg g^−1^; in *P. sarmentosa* they were between 50 and 100 µg g^−1^, and between 70 and 190 µg g^−1^ in *F. kinabaluensis* (Table 3), whereas Ni was predominantly concentrated in the phloem of *F. kinabaluensis* and *A. alanbakeri*. It also showed high enrichment in the epidermis of *A. alanbakeri* and *F. kinabaluensis* and in some parts of *P. sarmentosa* (Fig. 2; **see**  **Supporting Information—Figs S1**  **and**  **S2**).

The concentrations of Cl in the roots of *A. alanbakeri* ranged between 3060 and 6300 µg g^−1^, between 750 and 4070 µg g^−1^ in *P. sarmentosa* and between 670 and 1780 µg g^−1^ in *F. kinabaluensis* (Table 3) with enrichment in the cortex of *A. alanbakeri* and *F. kinabaluensis*, whereas in *P. sarmentosa* it was concentrated in the cortex and phloem (Fig. 2; **see**  **Supporting Information—Figs S1**  **and**  **S2**). The concentrations of K in *A. alanbakeri* were between 9460 and 14 800 µg g^−1^, whereas in *P. sarmentosa* it ranged from 3620 to 4600 µg g^−1^ and between 5600 and 9110 µg g^−1^ in *F. kinabaluensis* (Table 3) with strong enrichment in the cortex and phloem of all three species (Fig. 2; **see**  **Supporting Information—Figs S1**  **and**  **S2**). It was much more concentrated in the phloem of *A. alanbakeri* in comparison with the two other species **[see**  **Supporting Information—Fig. S2****]**. The concentrations of Ca in *A. alanbakeri* ranged from 345 to 890 µg g^−1^, in *P. sarmentosa* from 900 to 2030 µg g^−1^, and from 740 to 1300 µg g^−1^ in *F. kinabaluensis* (Table 3) with high enrichment in the cortex and phloem of *A. alanbakeri* and *F. kinabaluensis* and in some parts of the phloem of *P. sarmentosa* (Fig. 2; **see**  **Supporting Information—Figs S1**  **and**  **S2**).

Manganese concentrations in the roots of *A. alanbakeri* were between 30 and 55 µg g^−1^, between 10 and 30 µg g^−1^ in *P. sarmentosa* and between 6 and 65 µg g^−1^ in *F. kinabaluensis* (Table 3), with strong enrichment in the epidermis and cortex of *A. alanbakeri* and *F. kinabaluensis*, but low and evenly spread in *P. sarmentosa* (Fig. 2; **see**  **Supporting Information—Figs S1**  **and**  **S2**). The concentrations of Co in roots of all the three species were low with comparable values; in *A. alanbakeri* they ranged between 6 and 20 µg g^−1^, did not exceed 15 µg g^−1^ in *P. sarmentosa* and were between 6 and 15 µg g^−1^ in *F. kinabaluensis* (Table 3). Cobalt was enriched in the epidermis, cortex and phloem of *A. alanbakeri* and in the epidermis and cortex of *F. kinabaluensis*, but more evenly spread throughout the sections of *P. sarmentosa* (Fig. 2; **see**  **Supporting Information—Figs S1**  **and**  **S2**).

#### Old stems.

The concentration of Ni was 700 µg g^−1^ in the old stem of *A. alanbakeri*, 250 µg g^−1^ in *P. sarmentosa* and between 100 and 500 µg g^−1^ in *F. kinabaluensis* (Table 4), with enrichment in the cortex and phloem of *F. kinabaluensis* and *A. alanbakeri* and in the pith and xylem of *P. sarmentosa* (Fig. 3; **see**  **Supporting Information—Fig. S3**).

The concentration of Cl in the old stem of *A. alanbakeri* was 1670 µg g^−1^, whereas in *P. sarmentosa* it was 30 000 µg g^−1^ and between 520 and 540 µg g^−1^ in *F. kinabaluensis* (Table 4) with enrichment in the cortex of *A. alanbakeri* and *F. kinabaluensis* and in the cortex, phloem and xylem of *P. sarmentosa* (Fig. 3; **see**  **Supporting Information—Fig. S3**). The K concentration in *P. sarmentosa* was 27 400 µg g^−1^, whereas in *A. alanbakeri* it was 11 100 µg g^−1^ and between 7720 and 8230 µg g^−1^ in *F. kinabaluensis* (Table 4) with the highest enrichment in the cortex and phloem of *F. kinabaluensis* and *A. alanbakeri* (Fig. 3; **see**  **Supporting Information—Fig. S3**). In *P. sarmentosa*, this element was more evenly spread and showed enrichment in the part of xylem (Fig. 3). The concentration of Ca in old stem of *A. alanbakeri* was 1800 µg g^−1^ and 2440 µg g^−1^ in *P. sarmentosa*. In *F. kinabaluensis* its concentration ranged from 2340 to 2960 µg g^−1^ (Table 4), with enrichment in the cortex and phloem of *A. alanbakeri* and some ‘dots’ of enrichment in the cortex, phloem and xylem of *P. sarmentosa*, whereas in *F. kinabaluensis* it was more concentrated in the phloem (Fig. 3; **see**  **Supporting Information—Fig. S3**).

Manganese concentrations in the old stems of *F. kinabaluensis* were between 15 and 100 µg g^−1^, 60 µg g^−1^ in *A. alanbakeri* and 20 µg g^−1^ in *P. sarmentosa* (Table 4) with Mn enriched in the cortex of *A. alanbakeri*, and *F. kinabaluensis* while it was much more evenly spread in the cortex, xylem and phloem of *P. sarmentosa* (Fig. 3; **see**  **Supporting Information—Fig. S3**). The concentration of Co in *F. kinabaluensis* was between 4 and 14 µg g^−1^, whereas in *A. alanbakeri* it was 3 µg g^−1^ and below the limit of detection (<5 µg g^−1^) in *P. sarmentosa* (Table 4). In *F. kinabaluensis* there was a clear enrichment of this element in the cortex and phloem, while in *A. alanbakeri* there was some enrichment in the epidermis, in comparison with the other tissue parts (Fig. 3; **see**  **Supporting Information—Fig. S3**).

#### Young stems.

The Ni concentration in the young stem of *A. alanbakeri* was 1160 µg g^−1^, whereas in *G. brunneum* it was 175 µg g^−1^, 190 µg g^−1^ in *P. sarmentosa* and 100 µg g^−1^ in *F. kinabaluensis* (Table 5). In *A. alanbakeri*, there was a strong Ni enrichment in the phloem and relatively low enrichment in the pith and primary xylem (Fig. 4). In *G. brunneum*, Ni was more evenly spread throughout the whole stem section, with slight enrichment in the pith and xylem (Fig. 4). The enrichment in the pith and xylem was much more pronounced in *P. sarmentosa* (Fig. 4); this pattern was also visible in *F. kinabaluensis* (Fig. 4), but less clear because of overall lower concentration of Ni in the measured section.

The concentration of Cl in young stem of *A. alanbakeri* was 1600 µg g^−1^, whereas in *G. brunneum* it was 13 300 µg g^−1^, 6900 µg g^−1^ in *P. sarmentosa* and 700 µg g^−1^ in *F. kinabaluensis* (Table 5) with Cl concentrated in the cortex of *A. alanbakeri*, *P. sarmentosa*, *F. kinabaluensis* and in the cortex, phloem and xylem of *G. brunneum* (Fig. 4). The highest concentration of K in the young stems was in *G. brunneum*, where it reached 21 400 µg g^−1^, whereas in *A. alanbakeri* it was 11 700 µg g^−1^, 13 500 µg g^−1^ in *P. sarmentosa* and 13 600 µg g^−1^ in *F. kinabaluensis* (Table 5) with strong enrichment in the cortex, phloem, xylem and pith of *A. alanbakeri*, in the cortex and phloem of *G. brunneum*, *P. sarmentosa* and *F. kinabaluensis*, and some enrichment in the xylem and phloem of *P. sarmentosa* (Fig. 4). There was significant depletion of this element in the pith of *G. brunneum* in comparison with the other species. The concentration of Ca in young stem of *A. alanbakeri* was 2950 µg g^−1^, 2570 µg g^−1^ in *F. kinabaluensis*, 1540 and 1370 µg g^−1^ in *P. sarmentosa* (Table 5). Many small ‘dots’ (Ca-oxalate crystals) were visible in the pith and phloem of *P. sarmentosa* and *F. kinabaluensis*. In *P. sarmentosa*, there was an overall enrichment in the epidermis, cortex and phloem, whereas in *A. alanbakeri*, it was more concentrated in the epidermis, phloem and pith (Fig. 4).

The concentrations of Mn were very low in all studied species, at the 15–30 µg g^−1^ level with the exception of *A. alanbakeri*, where the average value for the whole section was 155 µg g^−1^ (Table 5), with enrichment in the epidermis, cortex and phloem (Fig. 4). The concentrations of Co were even lower, below the limits of detection with the exception of *A. alanbakeri* where Co was found at the 3 µg g^−1^ level (Table 5).

#### Leaves.

The concentrations of Ni in the leaves of *G. brunneum* were 1060 µg g^−1^, the only one species where the hyperaccumulation threshold was exceeded in the leaves’ sections; between 270 and 740 µg g^−1^ in *A. alanbakeri*, from 60 to 250 µg g^−1^ and from 85 to 360 µg g^−1^ in *P. sarmentosa* and *F. kinabaluensis*, respectively (Table 6). Nickel was mainly distributed in the lower and upper epidermal cells of the leaves of all the studied species. However, in *A. alanbakeri*, Ni was also strongly enriched in the spongy and palisade mesophyll (Fig. 5; **see**  **Supporting Information—Fig. S5**). Nickel concentration was lower in the phloem and xylem of all of the species (Fig. 5; **see**  **Supporting Information—Figs S4**  **and**  **S5**).

The concentration of Cl in *A. alanbakeri* ranged between 4200 and 4620 µg g^−1^, 17 300 µg g^−1^ in *G. brunneum*, between 12 400 and 16 400 µg g^−1^ in *P. sarmentosa* and from 1100 to 4300 µg g^−1^ in *F. kinabaluensis* (Table 6) with enrichment in the xylem of *A. alanbakeri*, spongy mesophyll of *G. brunneum*, upper and lower epidermis and palisade mesophyll of *P. sarmentosa* and xylem and phloem of *F. kinabaluensis* (Fig. 5; **see**  **Supporting Information—Figs S4**  **and**  **S5**). Potassium concentrations in the leaves of *G. brunneum* were 38 200 µg g^−1^, between 12 800 and 15 800 µg g^−1^ in *A. alanbakeri*, between 2650 and 10 300 µg g^−1^ in *F. kinabaluensis* and from 2300 to 7800 µg g^−1^ in *P. sarmentosa*. In all of the studied species, K was strongly enriched in the spongy and palisade mesophyll. There was also enrichment of K in the phloem of *F. kinabaluensis* and *G. brunneum* and in the xylem of *P. sarmentosa* (Fig. 5; **see**  **Supporting Information—Figs S4**  **and**  **S5**). Calcium in *A. alanbakeri* was between 700 and 6800 µg g^−1^, 3340 µg g^−1^ in the leaves of *G. brunneum*, between 900 and 3470 µg g^−1^ in *F. kinabaluensis* and between 690 and 1970 µg g^−1^ in the leaves of *P. sarmentosa* (Table 6) with enrichment in the spongy and palisade mesophyll of *A. alanbakeri* and *G. brunneum* and also in the upper and lower epidermis of *G. brunneum*, and in palisade mesophyll and phloem of *F. kinabaluensis* (Fig. 5; **see**  **Supporting Information—Figs S4**  **and**  **S5**). Small ‘dots’ highly enriched in Ca were also visible in the spongy and palisade mesophyll of *P. sarmentosa* (Fig. 5; **see**  **Supporting Information—Fig. S4**).

The concentrations of Mn in *A. alanbakeri* were between 90 and 390 µg g^−1^, whereas in *G. brunneum* it was 110 µg g^−1^ and ranged from 25 to 120 µg g^−1^ in *P. sarmentosa* and between 4 and 15 µg g^−1^ in *F. kinabaluensis* (Table 6) with some enrichment in the spongy and palisade mesophyll of *A. alanbakeri* and in the upper epidermis of *F. kinabaluensis* (Fig. 5). Cobalt concentrations in the leaves of all the studied species were low (Table 6) with some ‘dots’ of Co enrichment visible in the epidermis and spongy mesophyll of *A. alanbakeri* and *F. kinabaluensis* (Fig. 5; **see**  **Supporting Information—Figs S4**  **and**  **S5**).

### Scanning electron microscopy

**Figure 6.**
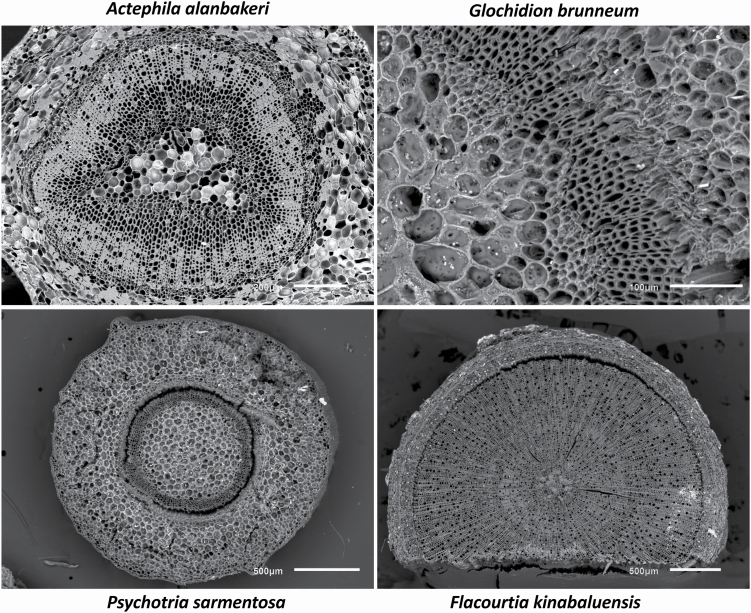
Scanning electron microscopy images of freeze-dried petiole cross-sections of *Actephila alanbakeri*, *Psychotria sarmentosa*, *Glochidion brunneum* and *Flacourtia kinabaluensis*.

The SEM images show that the freeze-dried petioles of *A. alanbakeri*, *P. sarmentosa*, *F. kinabaluensis* and *G. brunneum* have thick cuticle, multiseriate epidermis, and closely intact xylem and phloem (Fig. 6). This visually confirms that the very slow and low-temperature lyophilization process has left the (sub)cellular structures intact for the micro-PIXE analysis, and that elemental redistribution or other sample degradation is highly unlikely.

## Discussion

The plant tissue elemental concentrations reported in this study originate from samples collected from wild populations of *P. sarmentosa*, *G. brunneum*, *A. alanbakeri*, and *F. kinabaluensis* growing on ultramafic soil at Kinabalu Park. In comparison, the plant tissue samples used for the nuclear microprobe investigations originated from plants grown on ultramafic soil in a horticultural setting (Hyperaccumulator Botanical Garden) at Kinabalu Park. The ultramafic potting soil (Mollic Leptosol Hypermagnesic) contains far lower concentrations of plant-available Ni than the ultramafic soil (Hypermagnesic Cambisol) from the native populations (van der Ent *et al.* 2017b). In combination with the small pot size (2- to 3-L), this resulted in relatively low (<3000 µg g^−1^) foliar Ni concentrations compared to wild material (>10 000 µg g^−1^). This finding was unexpected, but has since been observed in a dedicated experiment on the effect of pot size on Ni hyperaccumulation in the temperate herb *Alyssum corsicum* (Chaney *et al.* 2017). Elements other than Ni may have also been affected, but conceivably only P and K, which are plant-essential macronutrients that are typically present in limited amounts in the ultramafic soils of Sabah (van der Ent *et al.* 2016).

The results from this study showed that the highest Ni concentrations are in the foliar epidermal cells of all four species. Similarly, in *Rinorea* cf*. bengalensis* and *P. rufuschaneyi*, Ni is also enriched in the foliar epidermal cells (van der Ent *et al.* 2017a). Boyd and Martens (1992) postulated that Ni enrichment in epidermal cells deters herbivory. However, Mesjasz-Przybyłowicz *et al.* (2016a) argued that this hypothesis would only make sense if symmetrical accumulation of Ni took place in both the upper and lower epidermis, as insect herbivores feed on both sides. Localization of Ni in epidermal cells has further been hypothesized to aid in osmoregulation and drought tolerance by increasing the water potential in the leaves (Severne 1974; Baker and Walker 1990; Boyd and Martens 1992; Mesjasz-Przybyłowicz *et al.* 1996). Preferential localization of Ni in the upper epidermal cell has also been suggested by Robinson *et al.* (2003) to act as a protection for the underlying chlorophyll against ultraviolet radiation. Nickel enrichment in the epidermal parts of leaves is a typical distribution pattern encountered in the majority of studied Ni hyperaccumulator plants to date from diverse phylogenetic and geographical affinities, e.g. *Senecio coronatus*, *S*. *anomalochrous* and *Berkheya zeyheri* subsp*. rehmannii* var*. rogersiana* (Mesjasz-Przybyłowicz *et al.* 1994, 1996, 2001) from South Africa; *Hybanthus floribundus* subsp. *floribundus* (Kachenko *et al.* 2008) and *Stackhousia tryonii* (Bhatia *et al.* 2004) from Australia; *Alyssum murale* (Broadhurst *et al.* 2004a; McNear *et al.* 2005), *A*. *bertolonii*, *A. lesbiacum* and *Noccaea goesingense* (Küpper *et al.* 2001) from Europe. An exception to this rule is *Berkheya coddii* (Asteraceae) where Ni is strongly enriched in the leaf veins and mesophyll, whilst the concentrations in the epidermis are relatively lower (Budka *et al.* 2005; Mesjasz-Przybyłowicz and Przybyłowicz 2011, 2020).

Different species of the Ni hyperaccumulator, *Alyssum*, have been reported to have Ni concentrated along with other elements in certain plant tissues. Accumulation of Ni, Mn and Ca was reported by Broadhurst *et al.* (2004b) at the base of *Alyssum* leaf trichomes. Subsequent studies by Broadhurst *et al.* (2009) also revealed a concentration of Ni and Mn only in trichome bases and in cells adjacent to the trichomes of *A. murale* and *A. corsicum*. In this study, the epidermal distribution of Ni as observed in the studied species is similar to that of the distribution of Ca and Cl in *P. sarmentosa* and *G. brunneum*, respectively. Simultaneous accumulation of other potentially toxic trace elements in plant tissues other than Ni such as Mn, Zn and Co has further been suggested by Boyd (2012) to deter herbivory.

Moreover, this study also revealed similar distribution patterns of K in the spongy and palisade mesophyll of the studied species. Calcium was also found enriched in the spongy and palisade mesophyll of *G. brunneum*, whereas in *A. alanbakeri*, both Ca and Ni were strongly enriched in the spongy and palisade mesophyll. This pattern of distribution may be explained by the essential requirement for K, Ca and Ni as plant nutrients by the studied species. Some Mn enrichment in the spongy and palisade mesophyll of *A. alanbakeri* and in the upper epidermis of *F. kinabaluensis* was also found in this study. On the other hand, Mn was found sequestered in the palisade mesophyll cells of the Mn hyperaccumulators *Gossia bidwillii*, *Virotia neurophylla*, *Macadamia integrifolia* and *M. tetraphylla* (Fernando *et al.* 2006a, b) where the authors indicated this to be due to the species high demand for Mn as part of the active centre of the oxygen-evolving complex. In *G. fragrantissima*, Co and Zn were found primarily localized in foliar epidermal cells whilst Mn and Ni were concentrated in the palisade layer (Fernando *et al.* 2013). Previous studies by Brooks *et al.* (1981) and Bidwell *et al.* (2002) have revealed that the Mn hyperaccumulators *Alyxia* sp. and *G. bidwillii* accumulate Mn at the expense of K and Mg. Cobalt distribution in *A. murale* was concentrated in the apoplast, which forms a Co-rich mineral precipitates on the foliar surface (Tappero *et al.* 2007). In comparison for *Glochidion* cf. *sericeum*, Co exudate was reported on the leaf surface in the form of lesions (van der Ent *et al.* 2018), where it was argued by the later authors to be due to the exposure of aerial oxygen that consequently led to oxidation of Co^2+^ to Co^3+^ on their leaf surfaces. However, in this study, minor Co was observed in the epidermis and spongy mesophyll of *A. alanbakeri* and *F. kinabaluensis*.

The phloem bundles are important tissues of Ni accumulation for the woody hyperaccumulators, such as *P. balgooyi*, *P. rufuschaneyi* and *Rinorea* cf*. bengalensis*, with up to 169 g kg^−1^ Ni in the phloem sap in *P. balgooyi* (Mesjasz-Przybyłowicz *et al.* 2016a; van der Ent *et al.* 2017a). High concentrations of Ni in the phloem have also been reported in herbaceous plants such as *S. coronatus* (Mesjasz-Przybyłowicz *et al.* 1997, 2007), *A. murale* (McNear *et al.* 2005; Tappero *et al.* 2007), *B. coddii* (Orłowska *et al.* 2013) and *B. zeyheri* subsp*. rehmannii* var*. rogersiana* (Mesjasz-Przybyłowicz *et al.* 2016b). This aligns with the results of this study on *F. kinabaluensis*, *P. sarmentosa* and *A. alanbakeri* with Ni enrichment in the phloem. In comparison with the New Caledonian Ni hyperaccumulator plants including *Homalium francii* (Phyllanthaceae), *Hybanthus austrocaledonicus* (Rubiaceae) and *P. gabriellae* (Salicaceae), Ni is also strongly localized in the epidermal cells and phloem bundles (Gei *et al.* 2020; Paul *et al.* 2020), and likewise in *Geissois pruinosa* (Cunoniaceae). However, *P. acuminata* (Sapotaceae) has Ni-rich laticifers, which constitute an independent network of cells parallel to the vascular bundles (Gei *et al.* 2020). In addition to the elevated concentrations of K, Ca, Cl, Mn and Co in the phloem of the studied species, these elements have also been found in the present study to be enriched in the cortex and epidermis of young stems, old stems and roots.

Even though Ni hyperaccumulation has ostensibly evolved numerous times independently in distant phylogenetic lineages in different areas around the world, the physiological mechanisms, as inferred from elemental localization, are convergent in these tropical woody species from Borneo Island.

## Supporting Information

The following additional information is available in the online version of this article—

**Figure S1.** Micro-PIXE elemental maps of *Flacourtia kinabaluensis* root section. Concentration scale in wt% dry weight or μg g^−1^ dry weight.

**Figure S2.** Micro-PIXE elemental maps of *Actephila alanbakeri* root section. Concentration scale in wt% dry weight or μg g^−1^ dry weight.

**Figure S3.** Micro-PIXE elemental maps of *Flacourtia kinabaluensis* old stem section.

**Figure S4.** Micro-PIXE elemental maps of *Psychotria sarmentosa* leaf section.

**Figure S5.** Micro-PIXE elemental maps of *Actephila alanbakeri* leaf section.

plaa058_suppl_Supplementary_Materials

## Notes

**Form & Function. Chief Editor:** Kate McCulloh
