# Supplementary material for: Convergent patterns of tissue-level distribution of elements in different tropical woody nickel hyperaccumulator species from Borneo Island
Source: AoB Plants. 2020 Nov 13;12(6):plaa058. doi: 10.1093/aobpla/plaa058 (PMC7759247; doi:10.1093/aobpla/plaa058)
Supplement: plaa058_suppl_Supplementary_Materials [file plaa058_suppl_supplementary_materials.pdf]

SUPPLEMENTARY INFORMATION

Convergent patterns of tissue-level distribution of elements in different tropical woody nickel hyperaccumulator species from Borneo Island

Farida Abubakari[1], Jolanta Mesjasz-Przybyłowicz[2],
Wojciech J. Przybyłowicz[2,3], Antony van der Ent[1,4*]

[1]Centre for Mined Land Rehabilitation, Sustainable Minerals Institute,
The University of Queensland, Australia.

[2]Department of Botany and Zoology, Stellenbosch University, Private Bag X1,
Matieland 7602, South Africa.

[3]AGH University of Science and Technology, Faculty of Physics & Applied Computer Science,
al. Mickiewicza 30, 30-059 Kraków, Poland.

[4]Université de Lorraine – INRA, Laboratoire Sols et Environnement, UMR 1120, France.

*Corresponding author: a.vanderent@uq.edu.au

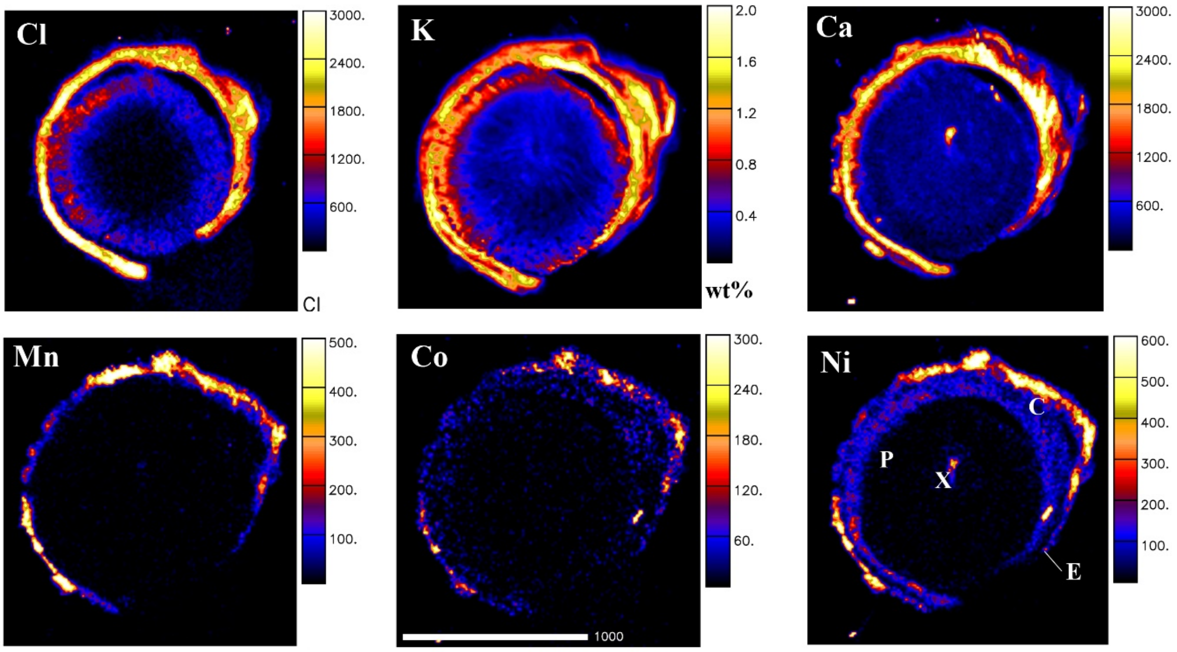

Supplementary Figure 1. Micro-PIXE elemental maps of *Flacourtia kinabaluensis* root section. Concentration scale in wt% dry weight or µg g^{-1} dry weight. Abbreviations of anatomical features: ***C*** cortex, ***E*** epidermis, ***P*** phloem and **X** xylem.

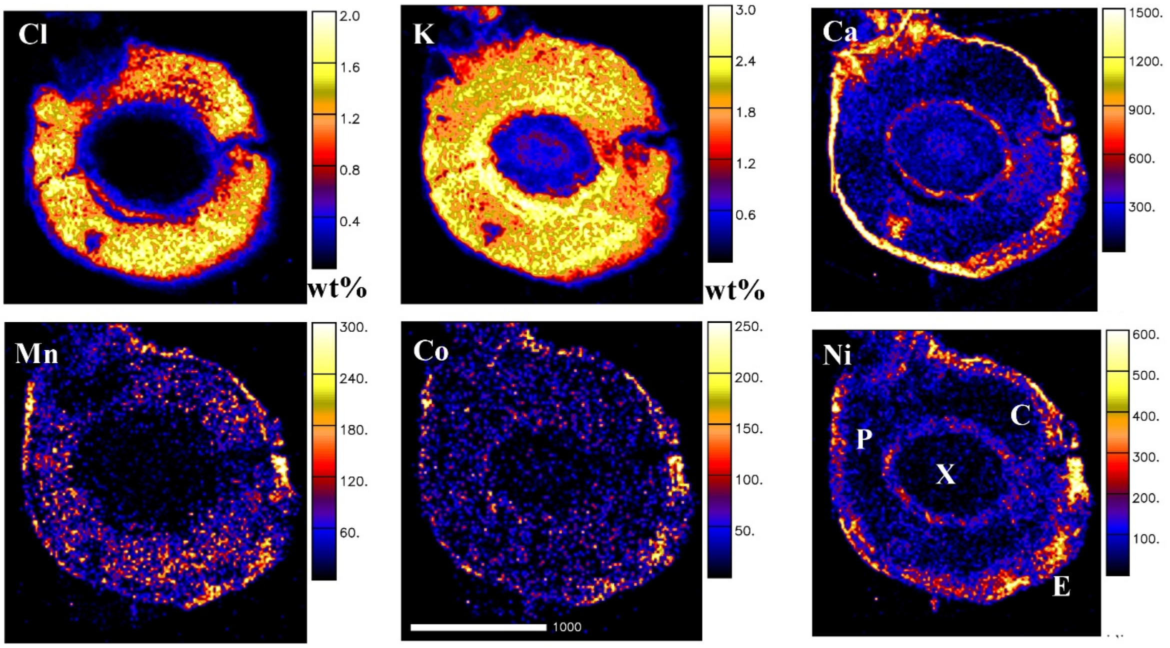

Supplementary Figure 2. Micro-PIXE elemental maps of *Actephila alanbakeri* root section. Concentration scale in wt% dry weight or µg g^{-1} dry weight. Abbreviations of anatomical features: ***C*** cortex, ***E*** epidermis, ***P*** phloem and **X** xylem.

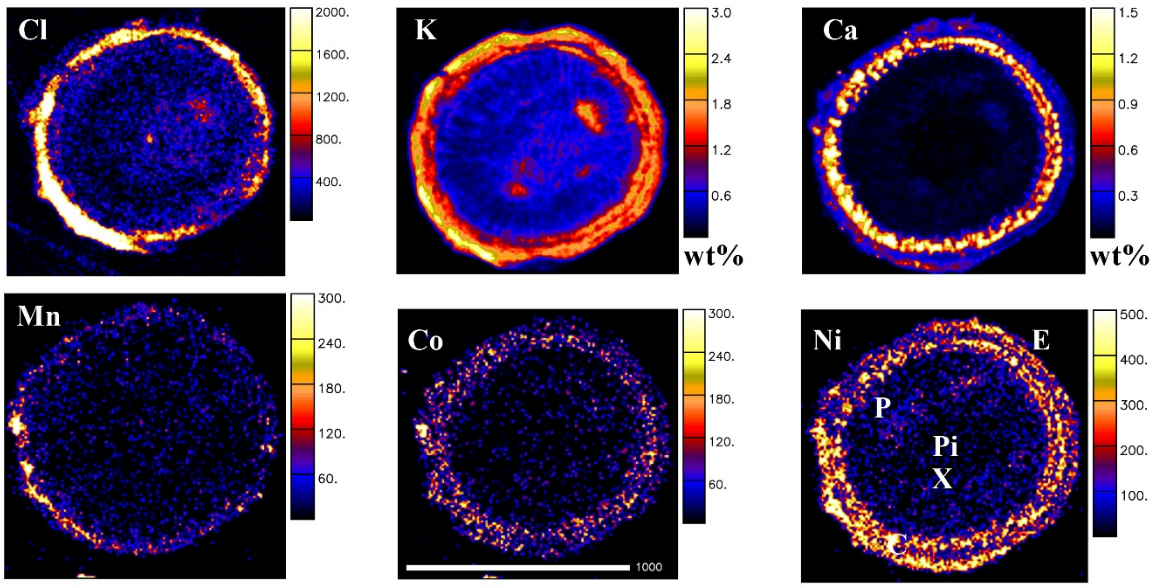

Supplementary Figure 3. Micro-PIXE elemental maps of *Flacourtia kinabaluensis* old stem section. Concentration scale in wt% dry weight or µg g^{-1} dry weight. Abbreviations of anatomical features: ***C*** cortex, ***E*** epidermis, ***Pi*** pith, ***P*** phloem and **X** xylem.

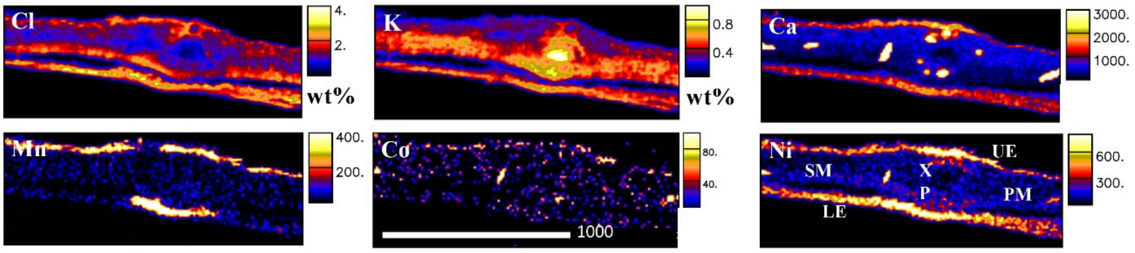

Supplementary Figure 4. Micro-PIXE elemental maps of *Psychotria sarmentosa* leaf section. Concentration scale in wt% dry weight or µg g^{-1} dry weight. Abbreviations of anatomical features: ***UE*** Upper epidermis, ***LE*** Lower epidermis, ***PM*** palisade mesophyll, ***SM*** spongy mesophyll, ***P*** phloem and ***X*** xylem.

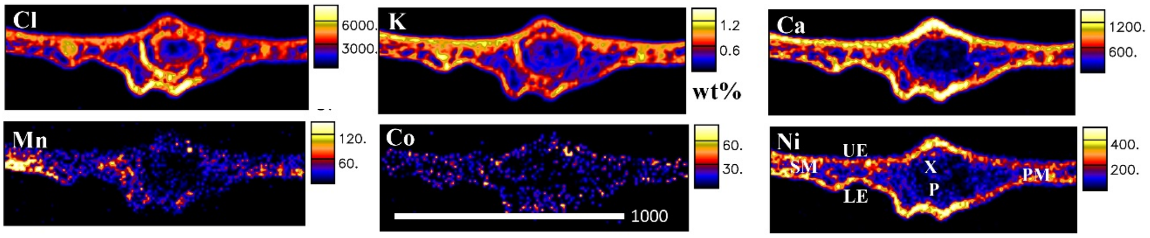

Supplementary Figure 5. Micro-PIXE elemental maps of *Actephila alanbakeri* leaf section. Concentration scale in wt% dry weight or µg g^{-1} dry weight. Abbreviations of anatomical features: ***UE*** Upper epidermis, ***LE*** Lower epidermis, ***PM*** palisade mesophyll, ***SM*** spongy mesophyll, ***P*** phloem and ***X*** xylem.
